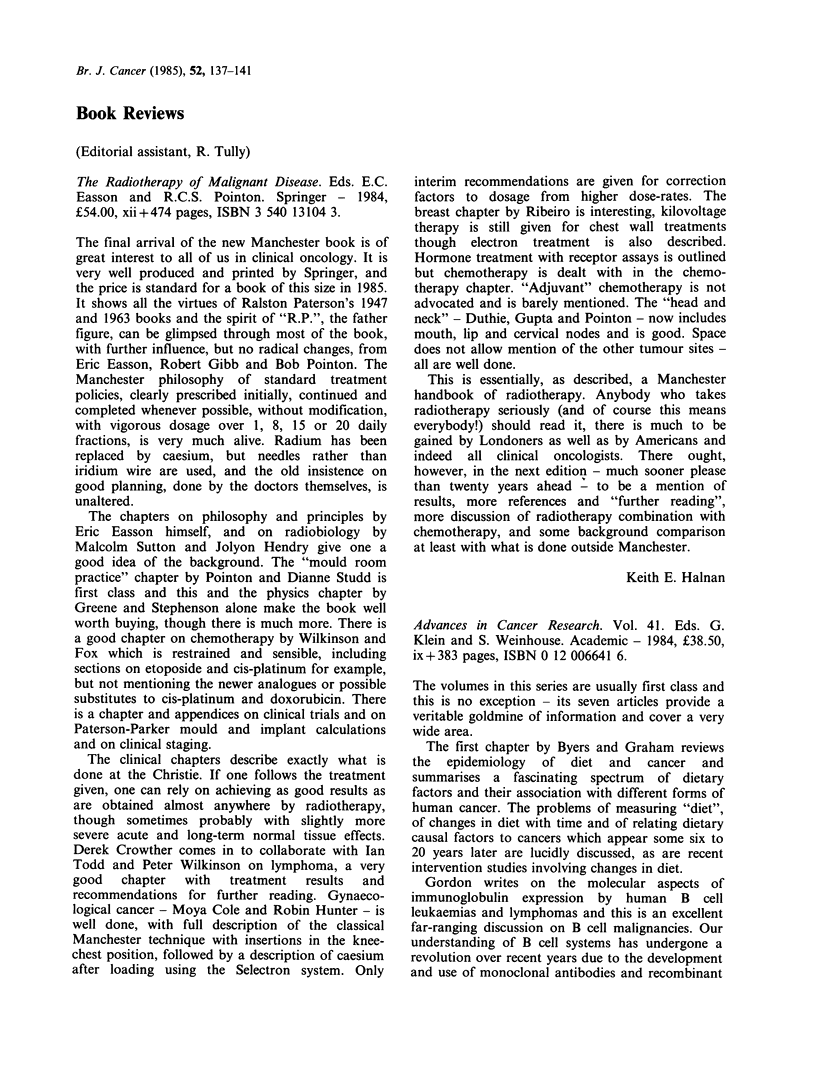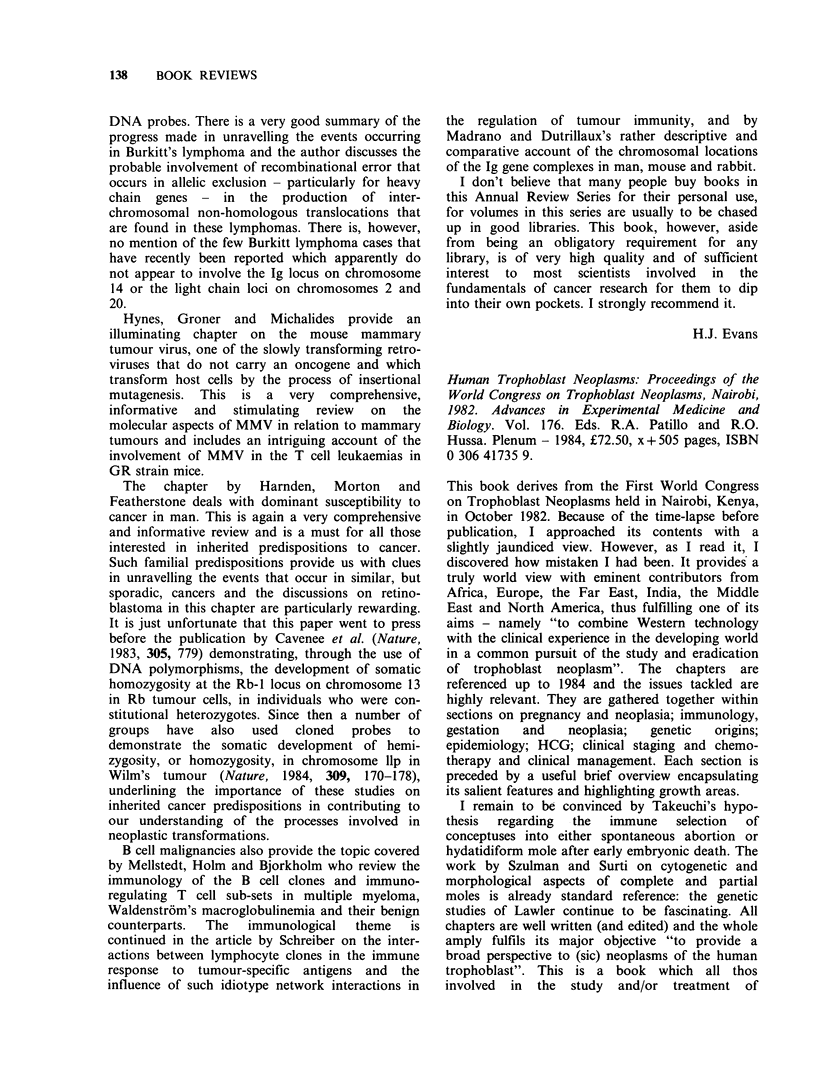# Advances in Cancer Research

**Published:** 1985-07

**Authors:** H.J. Evans


*Advances in Cancer Research*. Vol. 41. Eds. G. Klein and S. Weinhouse. Academic – 1984, £38.50, ix+383 pages, ISBN 0 12 006641 6.

The volumes in this series are usually first class and this is no exception – its seven articles provide a veritable goldmine of information and cover a very wide area.

The first chapter by Byers and Graham reviews the epidemiology of diet and cancer and summarises a fascinating spectrum of dietary factors and their association with different forms of human cancer. The problems of measuring "diet", of changes in diet with time and of relating dietary causal factors to cancers which appear some six to 20 years later are lucidly discussed, as are recent intervention studies involving changes in diet.

Gordon writes on the molecular aspects of immunoglobulin expression by human B cell leukaemias and lymphomas and this is an excellent far-ranging discussion on B cell malignancies. Our understanding of B cell systems has undergone a revolution over recent years due to the development and use of monoclonal antibodies and recombinant

DNA probes. There is a very good summary of the progress made in unravelling the events occurring in Burkitt's lymphoma and the author discusses the probable involvement of recombinational error that occurs in allelic exclusion – particularly for heavy chain genes – in the production of inter-chromosomal non-homologous translocations that are found in these lymphomas. There is, however, no mention of the few Burkitt lymphoma cases that have recently been reported which apparently do not appear to involve the Ig locus on chromosome 14 or the light chain loci on chromosomes 2 and 20.

Hynes, Groner and Michalides provide an illuminating chapter on the mouse mammary tumour virus, one of the slowly transforming retro-viruses that do not carry an oncogene and which transform host cells by the process of insertional mutagenesis. This is a very comprehensive, informative and stimulating review on the molecular aspects of MMV in relation to mammary tumours and includes an intriguing account of the involvement of MMV in the T cell leukaemias in GR strain mice.

The chapter by Harnden, Morton and Featherstone deals with dominant susceptibility to cancer in man. This is again a very comprehensive and informative review and is a must for all those interested in inherited predispositions to cancer. Such familial predispositions provide us with clues in unravelling the events that occur in similar, but sporadic, cancers and the discussions on retino-blastoma in this chapter are particularly rewarding. It is just unfortunate that this paper went to press before the publication by Cavenee *et al.* (*Nature,* 1983, **305,** 779) demonstrating, through the use of DNA polymorphisms, the development of somatic homozygosity at the Rb-1 locus on chromosome 13 in Rb tumour cells, in individuals who were con-stitutional heterozygotes. Since then a number of groups have also used cloned probes to demonstrate the somatic development of hemi-zygosity, or homozygosity, in chromosome llp in Wilm's tumour (*Nature,* 1984, **309,** 170–178), underlining the importance of these studies on inherited cancer predispositions in contributing to our understanding of the processes involved in neoplastic transformations.

B cell malignancies also provide the topic covered by Mellstedt, Holm and Bjorkholm who review the immunology of the B cell clones and immuno-regulating T cell sub-sets in multiple myeloma, Waldenström's macroglobulinemia and their benign counterparts. The immunological theme is continued in the article by Schreiber on the inter-actions between lymphocyte clones in the immune response to tumour-specific antigens and the influence of such idiotype network interactions in the regulation of tumour immunity, and by Madrano and Dutrillaux's rather descriptive and comparative account of the chromosomal locations of the Ig gene complexes in man, mouse and rabbit.

I don't believe that many people buy books in this Annual Review Series for their personal use, for volumes in this series are usually to be chased up in good libraries. This book, however, aside from being an obligatory requirement for any library, is of very high quality and of sufficient interest to most scientists involved in the fundamentals of cancer research for them to dip into their own pockets. I strongly recommend it.

H.J. Evans